# Uvaol Prevents Group B *Streptococcus*-Induced Trophoblast Cells Inflammation and Possible Endothelial Dysfunction

**DOI:** 10.3389/fphys.2021.766382

**Published:** 2021-12-03

**Authors:** Ana Lucia Mendes Silva, Elaine Cristina Oliveira Silva, Rayane Martins Botelho, Liliane Patricia Gonçalves Tenorio, Aldilane Lays Xavier Marques, Ingredy Brunele Albuquerque Costa Rodrigues, Larissa Iolanda Moreira Almeida, Ashelley Kettyllem Alves Sousa, Keyla Silva Nobre Pires, Ithallo Sathio Bessoni Tanabe, Marie-Julie Allard, Guillaume Sébire, Samuel Teixeira Souza, Eduardo Jorge Silva Fonseca, Karen Steponavicius Cruz Borbely, Alexandre Urban Borbely

**Affiliations:** ^1^Cell Biology Laboratory, Institute of Health and Biological Sciences, Federal University of Alagoas, Maceio, Brazil; ^2^Optics and Nanoscopy Group, Physics Institute, Federal University of Alagoas, Maceio, Brazil; ^3^Department of Pediatrics, McGill University, Montreal, QC, Canada; ^4^Department of Pediatrics, Université de Sherbrooke, Sherbrooke, QC, Canada; ^5^Faculty of Nutrition, Federal University of Alagoas, Maceio, Brazil

**Keywords:** Group B *Streptococcus*, infection, placenta, vascular dysfunction, trophoblast, uvaol

## Abstract

Group B *Streptococcus* (GBS) infection during pregnancy is involved in maternal sepsis, chorioamnionitis, prematurity, fetal infection, neonatal sepsis, and neurodevelopmental alterations. The GBS-induced chorioamnionitis leads to a plethora of immune and trophoblast cells alterations that could influence endothelial cells to respond differently to angiogenic mediators and alter placental vascular structure and function in pregnant women. In this context, preventive measures are needed to reduce such dysfunctions. As such, we evaluated the effects of a non-lethal exposure to inactivated GBS on trophoblast cells and chorionic villi explants, and if the treatment with uvaol would mitigate these effects. The concentration of 10^6^ CFU of GBS was chosen since it was unable to reduce the HTR-8/SVneo cell line nor term chorionic villi explant viability. Raman spectroscopy of trophoblast cells showed significant alterations in their biochemical signature, mostly reverted by uvaol. GBS exposure increased HTR-8/SVneo cells IL-1β and IFN-γ production, phagocytosis, oxidative stress, and decreased trophoblast cell migration. The Ea.hy926 endothelial cell line produced angiopoietin-2, CXCL-8, EGF, FGF-b, IL-6, PlGF, sPECAM-1, and VEGF in culture. When co-cultured in invasion assay with HTR-8/SVneo trophoblast cells, the co-culture had increased production of angiopoietin-2, CXCL-8, FGF-b, and VEGF, while reduced sPECAM-1 and IL-6. GBS exposure led to increased CXCL-8 and IL-6 production, both prevented by uvaol. Chorionic villi explants followed the same patterns of production when exposed to GBS and response to uvaol treatment as well. These findings demonstrate that, even a non-lethal concentration of GBS causes placental inflammation and oxidative stress, reduces trophoblast invasion of endothelial cells, and increases CXCL-8 and IL-6, key factors that participate in vascular dysregulation observed in several diseases. Furthermore, uvaol treatment prevented most of the GBS-provoked changes. Hence, uvaol could prevent the harmful effects of GBS infection for both the mother and the fetus.

## Introduction

Group B *Streptococcus* (GBS) or *Streptococcus agalactiae* is a gram-positive bacterium responsible for a great number of maternal and fetal morbidity and mortality. This bacterium is part of the vaginal and/or gastrointestinal tract of 10–30% of pregnant women, where the colonization is usually asymptomatic. Nevertheless, GBS can cause chorioamnionitis, and it is involved in the development of maternal and neonatal sepsis, prematurity, and alterations in fetal neurodevelopment (van Dillen et al., 2010; Allard et al., 2018; Doster et al., 2018; Zhu et al., 2019). Group B *Streptococcus* infection is also involved in endothelial dysfunction (Beyrich et al., 2011; Doster et al., 2018), which is known to be linked to other harmful conditions for both the mother and the fetus, such as preeclampsia (Valencia-Ortega et al., 2019). Due to the severity of GBS infection, the European consensus was achieved to recommend intrapartum antimicrobial prophylaxis based on a universal GBS screening strategy using fast real-time testing (di Renzo et al., 2015). Recently, the American College of Obstetricians and Gynecologists (ACOG) has also published updated guidelines to recommend GBS screening and prophylaxis, which replaced the previous guidelines from 2010 (Prevention of Group B Streptococcal Early-Onset Disease in Newborns | ACOG, 2010). Nonetheless, Latin American countries still do not have effective guidelines, and a recent study has shown that less than 15% of pregnant women are, indeed, screened for GBS, except Uruguay, where 65% of them are screened. In the latter country, GBS colonization was found in 18.5% of women, while the highest prevalence was found in black women, older women, and women without primary education (HogenEsch et al., 2021). Considering these data, it is important to highlight the deficiency of data for most Latin American countries. As an example, the biggest and more populated country of the region, Brazil, does not even have GBS screening and prophylaxis guidelines consensus in its National Healthcare Public System (Nascimento et al., 2019).

Although prophylaxis with antibiotics is proven effective (Vornhagen et al., 2017), a preventive alternative is inexistent at the moment. In this context, one of the biggest advances in obstetrics was the introduction of nutraceuticals to prevent fetal malformations or maternal conditions. A healthy diet is linked to reduced numbers of non-desirable pregnancy outcomes, such as abortion, prematurity, preeclampsia, gestational *diabetes mellitus*, intrauterine growth restriction (IUGR), and others (Wattar et al., 2019). Amongst all diets, it is well-known the benefits from the Mediterranean diet on poor pregnancy outcomes, particularly the ingestion of olives (*Europea olea*) or olive oil, which are potent anti-inflammatory foods (de La Torre et al., 2019; Wattar et al., 2019). One of its compounds, the pentacyclic triterpene uvaol (urs-12-ene-3,28-diol), is described to prevent trophoblast cell death, cytoskeleton changes-reduced membrane elasticity, and the increase of IL-1β, IL-2, and IFN-γ from GBS at 10^8^ CFU incubation (Botelho et al., 2019). Moreover, uvaol was also described to improve cell migration and tube formation in endothelial cells through PKA and p38-MAPK signaling pathways, increasing wound healing and angiogenesis (Carmo et al., 2020).

Hence, we believe that GBS-induced chorioamnionitis could lead to a plethora of immune and trophoblast cells alterations, which, in turn, influence endothelial cell function in pregnant women. As such, we evaluated the effects of a non-lethal exposure of inactivated GBS on trophoblast cells and chorionic villi explants, the repercussions on endothelial cells, and if the treatment with uvaol would mitigate these effects.

## Materials and Methods

### Cell Culture

The first trimester-derived extravillous trophoblast cell line HTR-8/Svneo, and the endothelial cell line EaHy-926 was separately cultured in the DMEM/F12 medium (Merck/Sigma-Aldrich, St. Louis, MO, United States) with 10% fetal bovine serum (FBS) and 2-mM L-glutamine (all from Thermo Fisher Scientific, Waltham, MA, United States) at 37°C and 5% CO_2_. HTR-8/SVneo cells were subcultured when ∼70% confluence was achieved, whereas EaHy-926 with ∼90% confluence. Cells were reseeded at 10^4^ cells/cm^2^.

### Collection and Culture of Placental Chorionic Villi Explants

The samples were collected at the Obstetrics Service of HUPAA/UFAL from healthy term pregnancies (37–40 weeks) with cesarean deliveries, under an approved ethics committee protocol. A total of eight placentas were obtained from 18- to 35-year-old women, with no current gestational, autoimmune, and infectious diseases, and genetic disorders. The study was approved by the Ethics Committee (52237915.5.0000.5013), and ethical considerations were based on the use of the material for scientific purposes, with the confidentiality of the patient identity and without constraint from the institutions or people involved. After the placental collection, two cotyledons were washed with saline solution and carefully scraped with a scalpel blade to obtain the terminal villi in a glass Petri dish. Samples were grown in the DMEM/F12 medium, and, after 24 h, the media was renewed.

### Uvaol Treatment

Uvaol (Merck/Sigma-Aldrich) was diluted in 1% dimethyl sulfoxide (DMSO) in phosphate-buffered saline (PBS) (v/v). Treatment with uvaol or only its vehicle was performed 24 h from the cell plating. The chosen concentration for HTR-8/SVneo cells was 10 μM, which we previously showed as a concentration that does not affect cell viability (Botelho et al., 2019), and, for placental explants, uvaol was used at 50 μM based on the following 3-(4,5-dimethylthiazol-2-yl)-2,5-diphenyltetrazolium bromide (MTT) results.

### Group B *Streptococcus* Incubation

The β-hemolytic GBS serotype Ia strain (#16955) that we used has had its isolation, growth, and inactivation methods described previously (Bergeron et al., 2013). Briefly, GBS was isolated at CHUS Fleurimont from a pregnant patient and grown in brain-heart infusion (BHI) broth for 15 h at 37°C. The culture was diluted at 1:100 and further grown until the mid-exponential phase. Titration was performed by counting colony-forming units (CFU) on BHI agar plates. The bacteria were harvested by centrifugation at 3,220 g and inactivated in a solution of 10% formaldehyde for 24 h. Afterward, GBS was washed thoroughly with saline solution. Inactivated GBS was harvested and suspended in sterile suspension at 10^9^ CFU/100 μL; 50 μL of the inactivated suspension was plated in duplicate on BHI agar and incubated at 37°C overnight to verify that all bacteria were killed. For the *in vitro* incubation experiments, GBS at 10^6^ CFU/ml was used. In experiments of cell death, *in vitro* wound healing assay, and 3D co-culture endothelial invasion test, GBS was incubated for 48 h. Some phagocytosis assays had GBS incubation for 2 h. Other experiments had 24 h incubation time.

### The MTT Cell Viability Assay

The 3-(4,5-dimethylthiazol-2-yl)-2,5-diphenyltetrazolium bromide (MTT) assay was used to verify the possible cytotoxicity of uvaol and GBS. As such, HTR-8/SVneo cells or placental explants were treated with uvaol for 1 h before GBS incubation or untreated, followed by GBS incubation, and further cultured in DMEM-F12 media supplemented with 10% FBS for 24 h. The medium was replaced with a fresh culture medium, containing 5 mg/ml of MTT, and the supernatant was discarded after a 4-h incubation period at 37°C, followed by the addition of DMSO solution. The absorbance of the solubilized MTT formazan product was spectrophotometrically measured at 540 nm. The percentage viability was determined about control [(absorbance of treated cells/absorbance of untreated cells) × 100].

### Measure of Cell Death

Cell death was evaluated using the Annexin V-fluorescein (FITC)/Propidium Iodide (PI) detection kit (BD Biosciences) according to the descriptions of the manufacturer. In summary, 3 × 10^5^ cells were plated, and the cells were analyzed after 24 h and 48 h after uvaol treatment and GBS incubation. After cell detachment with 25% trypsin solution (v/v), cells were incubated with BD Binding Buffer (BD Biosciences, Franklin Lakes, NJ, United States) and then incubated with 5 μL of annexin V and 5 μL of PI in the dark for 20 min. The results were acquired with the flow cytometer BD FACS Canto II (BD Biosciences) and analyzed using the software FlowJo v.10.7.2 (BD Biosciences).

### Raman Spectral Measurements

Raman spectra were measured using an XploRA spectrometer (Horiba, Japan), coupled to an optical microscope (BXFM, Olympus, Japan) and equipped with a 532-nm laser that was focused on the nucleus of the cells through a 100 × objective (NA = 0.9). The same objective lens was used for collecting Raman-scattered light after interaction with the sample, in backscattering geometry. The frequency calibration was set by reference to the 520-cm^–1^ vibrational bands of a silicon wafer. Under the same conditions, 60 cell spectra captured in three different experiments for each group were measured in the spectral range of 600–1,800 cm^–1^. To minimize laser-induced heating of the specimens, low-power irradiation at the sample surface was used, around 5 mW, during a short exposure time (3-s laser exposure for five accumulations). The diffraction grating used had 1,200 lines/mm, which yielded a spectral resolution of 1.5 cm cm^–1^.

### Data Preprocessing and Spectral Analysis

Before conducting the spectral analysis, all spectra were smoothed, background-adjusted, and normalized using an algorithm implemented in MatLab software (Mathworks, Naticks, MA, United States). As such, the external noises were suppressed, and the useful information about the biochemical composition was enhanced. After removing the background fluorescence from the spectra, Principal Component Analysis (PCA) and Hierarchical Cluster Analysis (HCA) were performed to evaluate the spectral variability in the dataset. Each cell group was subjected to 3D-PCA and an unsupervised HCA with Euclidean distances. All multivariate statistical analyses were implemented in MatLab software. Orthogonal Partial Least Squares Discriminant Analysis (OPLS-DA) was used to analyze the spectral differences in the samples and to predict which class each sample belongs to. This multivariate statistical analysis was performed through the SIMCA 17 software (Umetrics, Umea, Sweden). Moreover, Raman individual peaks with statistically significant fluctuations were isolated to compare. For further analysis, the extracted band intensities were imported to GraphPad Prism software (Graph Pad Software Inc., San Diego, CA, United States) to apply one-way ANOVA and statistical analysis.

### Phagocytosis Assay

HTR-8/SVneo cells were cultured at 5 × 10^4^ in 24-well plates containing glass coverslips. After 24 h, they were treated with 10-μM uvaol for 1 h and stimulated with inactivated or live GBS at 10^6^ CFU for further 2 h. Afterward, cultures were washed with PBS, fixed with ice-cold methanol, and stained with Giemsa staining. The coverslips were mounted with Entellan (Sigma-Aldritch) on histological slides and examined under a Nikon Eclipse 50i optical microscope (Nikon, Japan) in a 100 × objective with immersion oil. The percentage of phagocytosis was determined by the presence of GBS inside trophoblast cells cytoplasm [(number of cells with GBS in the cytoplasm/total number of cells) × 100]. Additionally, groups to analyze the influence of recombinant addition of 100 UI/ml IFN-γ (Merck/Sigma-Aldrich; Albieri et al., 2005) on phagocytosis were performed where cells were treated for 24 h with IFN-γ before GBS incubation or 24 h after GBS incubation.

### Mitochondrial Reactive Oxygen Species Production Assay

After the previously described treatments, MitoSOX red (Thermo Fisher Scientific, Waltham, MA, United States) staining was incubated in living cells, following the conditions of the manufacturer. After 30 min, cells were fixed with 4% paraformaldehyde in PBS (v/v), washed with PBS, stained with 4’,6-diamidino-2-phenylindole (DAP-I), and mounted with PBS/glycerol (1:9, v/v) under glass slides. The results were visualized with a fluorescence microscope Nikon DS-Ri1 (Nikon). Images were acquired using the DP2-BSW software (Nikon). The percentage of cells with Mitochondrial Reactive Oxygen Species (mtROS) was determined by [(number of cells positively stained by MitoSOX/total number of cells) × 100].

### Cytoskeleton Evaluation of F-Actin Polymerization

HTR-8/SVneo cells were plated at 3 × 10^5^ cells, and, after 24 h, uvaol was added 1 h before GBS incubation and maintained for 24 h. The cells were then fixed with 4% paraformaldehyde in PBS and permeabilized with.1% Triton X-100 in PBS (v/v). Phalloidin-fluorescein (FITC)-conjugated staining (1:100; Abcam, Cambridge, United Kingdom) was added for 1 h, and nuclei were stained with DAP-I. Cells were mounted with PBS/glycerol (1:9, v/v) under glass slides, and the results were visualized with fluorescence microscope Nikon DS-Ri1 (Nikon). Images were acquired using the DP2-BSW software (Nikon).

### *In vitro* Wound-Healing Assay

HTR-8/SVneo cells were plated at 3 × 10^5^ cells until reaching ∼ 100% confluence. Afterward, scratches of equal size were performed with a 200-μL pipette tip. Cells were treated with 10-μg/ml mitomycin C (Sigma-Aldritch) for 2 h. Uvaol was added 1 h before GBS incubation and maintained for 48 h. Pictures were taken after 12, 24, 36, and 48 h (400 × magnification). The distance from the scratch borders was measured using the ImageJ free software (NIH, Bethesda, MD, EUA), and the closure percentage of each point was calculated.

### Three-Dimensional Vascular Invasion Assay

The Three-Dimensional (3D) vascular invasion assay was performed in transwell chambers using 24-well fitted inserts with 8-μm pore size. A mixture of 8 × 10^5^ EaHy-926 endothelial cells/well in 15 μL of Matrigel (Sigma; 1:1 v/v in DMEM/F12) was plated on the upper chamber, and DMEM/F12 was added in both compartments after polymerization. The cells were kept until ∼100% confluence was achieved. Afterward, HTR-8/SVneo cells were stained with 1 μM of carboxyfluorescein succinimidyl ester (CFSE, Thermo Fisher Scientific) and kept in the dark for 10 min. The CFSE-stained HTR-8/SVneo cells were placed at 2 × 10^5^ cells/well on top of the previous mixture of Matrigel and EaHy-926 cells, and supplemented DMEM/F12 was added in both compartments (5% FBS in an upper compartment with 10-μM uvaol, and 20% FBS in the lower compartment). After 1 h, GBS was added to the upper compartment. After 48 h, non-invading cells from the upper compartment were removed with a cotton swab, supernatants were collected, and the membranes were fixed with ice-cold methanol. Nuclei were stained with DAP-I, and the cut membranes were mounted with PBS/glycerol (1:9, v/v) under glass slides. The results were visualized with fluorescence microscope Nikon DS-Ri1 (Nikon, Japan), and images were acquired at 400 × magnification using the DP2-BSW software (Nikon).

### Supernatant Detection of Cytokines and Angiogenic Factors

All collected supernatants were analyzed for IL-1β using an ELISA kit (Sigma-Aldrich) following the instructions of the manufacturer. Tetramethylbenzidine and hydrogen peroxide were used as substrates in the peroxidase reaction, and plates were read at 450 nm using a Magpix system (Sigma-Aldrich). The supernatants from HTR-8/SVneo cells were evaluated using the LEGENDplex human Th1/Th2 Panel (BioLegend, San Diego, CA, United States) kit for detecting IL-2, IL-4, IL-5, IL-6, IL-10, IL-13, TNF-α, and IFN-γ levels. The supernatants from HTR-8/SVneo cells, the 3D vascular invasion assay, and from placental chorionic villi were analyzed with the LEGENDplex human Angiogenic Panel (BioLegend) kit for detecting IL-6, CXCL-8, Angiopoietin-1, Angiopoietin-2, fibroblast growth factor basic (FGF-b), epidermal growth factor (EGF), soluble PECAM-1 (sPECAM-1), placental growth factor (PlGF), vascular endothelial growth factor (VEGF), and TNF-α. Both LEGENDplex kits were employed according to the instructions of the manufacturer. The cytokines and angiogenic factors were detected using FACS Canto II (BD Biosciences), and the mean fluorescence intensity (MFI) from the samples was converted to pg/ml using the LEGENDplex v.8.0 software (BioLegend).

### Statistical Analysis

To show that all data were normally distributed, a Kolmogorov-Smirnov test was performed. Matlab software was used for spectral analysis, and PCA and HCA were used for spectral multivariate analysis. Classification of samples by OPLS-DA was directly reflected by the principal component scores of the OPLS-DA model, and the robustness of this model was assessed based on the following parameters: R2X (cum), the cumulative sum of squares of all x-variables explained by all extracted components; R2Y(cum), the cumulative sum of squares of all y-variables explained by all extracted components; and Q2(cum), the fraction of all x-variables and y-variables that can be predicted for the extracted component. Other results were analyzed using Graph Pad Prism (Graph Pad Software Inc., San Diego, CA, United States) with one-way ANOVA using Dunnett or Tukey *post hoc* test, and cell death was analyzed with two-way ANOVA using Bonferroni *post hoc* test. The minimal level of significance for all experiments was set at *p* < 0.05. The results are depicted as the mean ± standard error of the mean (SEM).

## Results

### Inactivated Group B *Streptococcus* Does Not Change Placental Explants and Trophoblast Cell Viability

**FIGURE 1:**
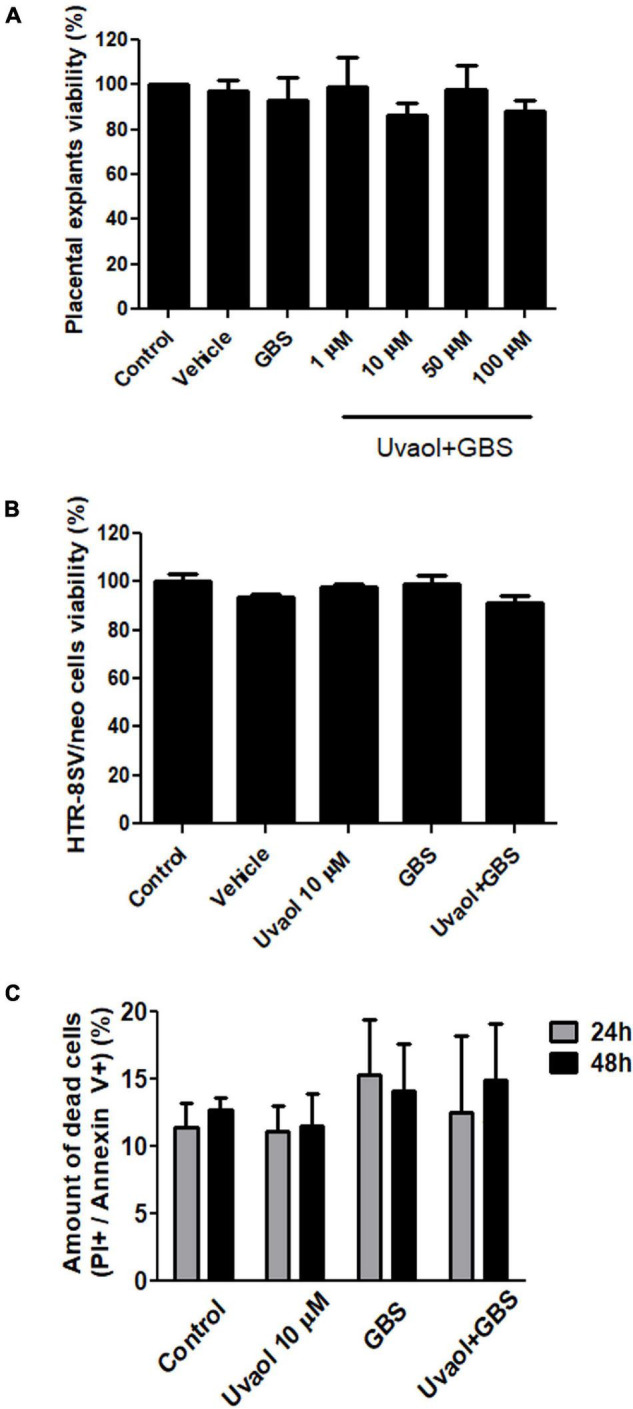
Uvaol does not affect cells and explant viability. **(A)** The term chorionic villi explants were treated with DMSO (vehicle), GBS at 10^6^ CFU (GBS), or with uvaol at 1, 10, 50, and 100 μM for 24 h and analyzed by MTT assay. **(B)** The HTR-8/SVneo cells were treated with vehicle, 10-μM uvaol, GBS at 10^6^ CFU or both for 24 h, and analyzed by MTT assay. **(C)** Cells were treated for 24 h and 48 h, and annexin V/PI staining was analyzed through flow cytometry. Bar graphs represent mean values ± S.E.M.; *n* = 4 in triplicate in panel **(A,B)**, and *n* = 3 in duplicate in panel **(C)**. One-way ANOVA with Tukey *post hoc* test was used in panel **(A,B)**, whereas two-way ANOVA with Bonferroni *post hoc* test was used in panel **(C)**.

Placental explants incubated with GBS at 10^6^ CFU alone or after uvaol treatment had no statistically significant changes in overall viability concerning control (Figure 1A). A similar result was found in HTR-8/SVneo cells, with no changes in their viability (Figure 1B). To assess if GBS at 10^6^ CFU, indeed, did not induce cell death, we analyzed the Annexin V and Propidium Iodide (PI) staining of trophoblast cells by flow cytometry. As result, no differences were observed, comparing all analyzed groups, nor when different time points (24 and 48 h) were evaluated (Figure 1C).

### Raman Spectra Classification and Analysis

**FIGURE 2:**
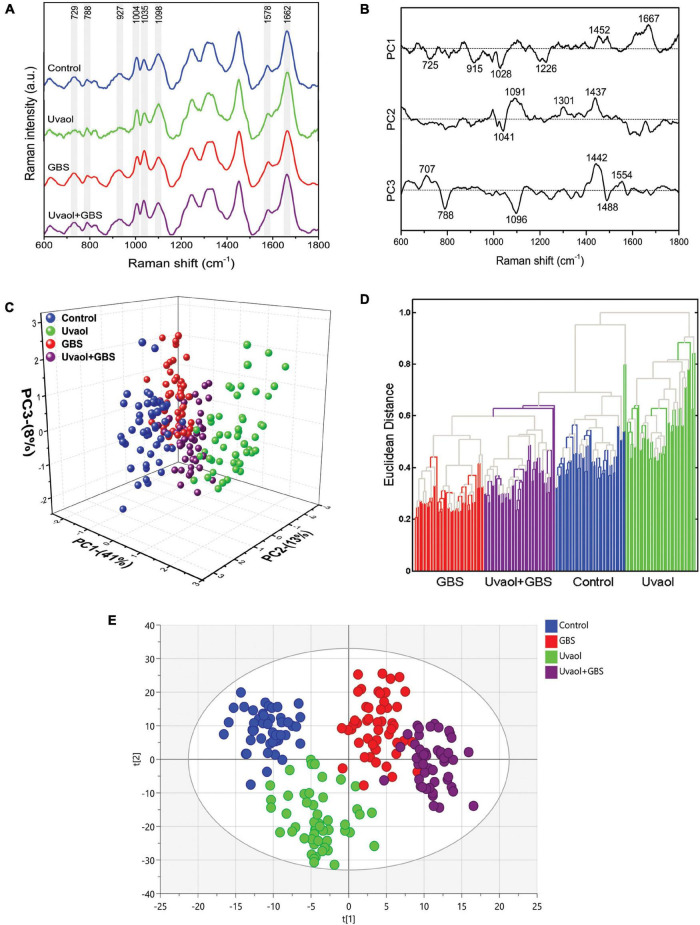
GBS and uvaol change trophoblast cells biochemical signature. **(A)** The Raman spectra represent the averages of 50 cells for each group (Control, uvaol 10 μM, GBS at 10^6^ CFU, and uvaol with GBS) in the fingerprint region (600–1,800 cm^–1^). The shaded areas correspond to the Raman bands where the main variances observed in the average cell spectra occurred; **(B)** Loadings of PC1, PC2, and PC3 for Control, uvaol, GBS, and uvaol + GBS groups; **(C)** A three-dimensional PCA score plot; **(D)** HCA dendrogram for all analyzed groups; **(E)** A two-dimensional OPLS-DA score plot of the Raman spectra for the Control, uvaol, GBS, and uvaol + GBS groups. The four groups were relatively well-discriminated along the t[1] axis, as well along the t[2] axis. *N* = 3.

Through the Raman spectra, it was possible to identify the most important spectral differences among trophoblast cell groups. To perform spectral data analysis and classification, the Raman spectra were divided into three groups: (i) cells without treatment (control); (ii) cells treated with uvaol (uvaol); (iii) cells incubated with GBS at 10^6^ CFU (GBS); and (iv) cells treated with uvaol and incubated with GBS (uvaol + GBS). A total of 60 cell spectra were measured for each group, from three independent experiments, and Figure 2A shows the average Raman spectra of the analyzed groups. We focused on all measurements over cell nuclei, even though Raman spectroscopy can be performed at any cell portion. Nonetheless, we found that HTR-8SV/neo cells were thin at the cell periphery, and the glass surface below greatly interfered with the readings. Moreover, the standardization of the region reduces variations that might be observed if random cell places were measured otherwise (Moor et al., 2018; Arend et al., 2020; Tiwari et al., 2020). A three-dimensional plot was constructed with combinations of sets of scores of the first three PCs as well as the corresponding plots of the PC1 and PC2 loadings, used for the determination of the differentiation capability of PCA and identification of significant Raman features (Figures 2B,C). The PC loadings are representative of the biochemical differences between cell groups and are responsible for differentiating the spectra in the score plot of PC. Indeed, the PC loadings effectively carry all the important information of the spectra and have a spectral dimension, where positive and negative peaks can be observed. In the PCA, loadings of PC1 indicated positive correlations of Raman bands at 1,452 and 1,667 cm^–1^, and negative correlations at 725, 915, 1,028, and 1,226 cm^–1^. The PC2 loadings indicated positive correlations in 1,091, 1,301, and 1,437 cm^–1^, whereas negative correlations at 1,041 cm^–1^. The PC3 loadings indicated positive correlations at 707, 1,442, and 1,554 cm^–1^, whereas negative correlations at 788, 1,096, and 1,488 cm^–1^ (Figure 2B). As such, the first three PCs explained 62% of the variance of the original data set, with PC1 describing 41%, PC2 describing 13%, and PC3 describing 8% of the total variance, with principal component analysis (PCA) clearly dividing cells into four different clusters matching their groups (Figure 2C). Furthermore, hierarchical cluster analysis (HCA) was used for cell discrimination, and four separated clusters could be easily observed, each one representing a group, distinctively aggregated, with the GBS group being the most distant from the control group (Figure 2D). The clusters showed a clear separation with no mixture of cells from different groups, corroborating the PCA and suggesting that uvaol, indeed, prevents certain biochemical changes induced by GBS (Figure 2D). We also employed the OPLS-DA, as it can separate predictive from non-predictive (orthogonal) variation to highlight the differences among the analyzed groups. The samples were separated with minimal overlap in the score scatter plot along the X and Y axes (Figure 2E) at a 95% confidence interval. The OPLS-DA of the Raman spectra resulted in the following specific parameters: R2X(cum) = 0.521, R2Y(cum) = 0.860, and Q2(cum) = 0.812 (*p* < 0.05). As such, the obtained parameters indicated that the OPLS-DA not only had discrimination power—they also indicated predictive precision of 86% to assign an unknown sample to one of the four analyzed groups.

### Raman Spectrum Assignment of Trophoblast Cells

Based on the most significant changes, Raman band assignments and contributions used in the interpretation of spectral features were based on the published literature (Gelder et al., 2007; Movasaghi et al., 2007; Başar et al., 2012; Talari et al., 2014; Czamara et al., 2015; Surmacki et al., 2015; González-Solís et al., 2018; Keleştemur et al., 2018; Balan et al., 2019; Casal-Beiroa et al., 2021) and detailed in Table 1. Analyzing only uvaol treatment on trophoblast cells, we could observe that the Raman bands in 725, 729, 915, 927, 1,004, 1,035, 1,041, 1,091, 1,226, 1,301, and 1,437 cm^–1^ had reduced intensities in comparison to the control group, whereas the 788, 1,028, 1,452, 1,488, 1,554, 1,578, 1,662, and 1,667 cm^–1^ had increased intensities. Regarding GBS group differences in comparison to control, the bands in 1,004, 1,091, 1,096, 1,098, 1,301, 1,437, 1,442, 1,488, 1,662, and 1,667 cm^–1^ were reduced, while the bands in 725, 729, 788, 915, 1,028, 1,035, 1,041, 1,226, and 1,578 were increased. Interestingly, several of the bands reduced or increased in the uvaol group had the opposite result in the GBS group, which indicates that uvaol produces several biochemical alterations that could prevent trophoblast cells from the GBS opposite effects, or maybe help these cells to counter the negative effects risen from GBS contact. Consonantly, when we compared the Uvaol + GBS group with the GBS group, from the 20 changed Raman bands, uvaol prevented the alterations from 11 bands (725, 729, 915, 1,004, 1,028, 1,041, 1,091, 1,096, 1,098, 1,662, and 1,667 cm^–1^), with a synergic effect of only one band (788 cm^–1^). Regarding the 11 bands that uvaol helped to maintain after GBS incubation, they were mainly related to DNA, RNA, phenylalanine, glutathione, glycosaminoglycans, myristic acid, membrane phospholipids, cholesterols, and amide I. Nevertheless, the bands in 1,035, 1,226, 1,301, 1,437, 1,442, 1,452, 1,488, and 1,578 cm^–1^ were mainly related to amide I, amide III, fatty acids, lipids, proteins, and purines, which were altered by the GBS, although uvaol treatment was not able to prevent these changes. Altogether, the spectral analysis of the described bands can lead to the observation that a non-lethal concentration of GBS strongly modifies the biochemical composition of trophoblast cells, while uvaol prevented half of these changes.

**TABLE 1:** Raman bands position and their respective assignments and contributions.

Raman band position [cm^–1^]	Band assignment	Contributions	Uvaol differences to control (*P*-values)	GBS differences to control (*P*-values)	Uvaol + GBS differences to GBS (*P*-values)
707	ν(C-S)	Methionine	NS	NS	NS
725	Ring breathing modes	Adenine	Reduced intensity (*p* < 0.001)	Increased intensity (*p* < 0.01)	Reduced intensity (*p* < 0.01)
729	Ring breathing modes	Adenine	Reduced intensity (*p* < 0.001)	Increased intensity (*p* < 0.01)	Reduced intensity (*p* < 0.01)
788	υ(O-P-O)	DNA	Increased intensity (*p* < 0.01)	Increased intensity (*p* < 0.01)	Increased intensity (*p* < 0.05)
915	βCH	RNA (Ribose)	Reduced intensity (*p* < 0.001)	Increased intensity (*p* < 0.001)	Reduced intensity (*p* < 0.01)
927	υ(C–C)	CoA, Proline, Valine	Reduced intensity (*p* < 0.001)	NS	NS
1004	Ring breathing modes	Phenylalanine	Reduced intensity (*p* < 0.001)	Reduced intensity (*p* < 0.001)	Increased intensity (*p* < 0.001)
1028	βCH	Phenylalanine	Increased intensity (*p* < 0.001)	Increased intensity (*p* < 0.001)	Reduced intensity (*p* < 0.001)
1035	υ(C-C)	Amide I	Reduced intensity (*p* < 0.001)	Increased intensity (*p* < 0.001)	NS
1041	υ(C-O-C)	Glycosaminoglycans, Glutathione	Reduced intensity (*p* < 0.001)	Increased intensity (*p* < 0.001)	Reduced intensity (*p* < 0.001)
1091	υ(C–C)	Myristic acid	Reduced intensity (*p* < 0.01)	Reduced intensity (*p* < 0.001)	Increased intensity (*p* < 0.001)
1096	υ_s_(O-P-O), (PO_2_^–^)	Membrane phospholipids, DNA	NS	Reduced intensity (*p* < 0.001)	Increased intensity (*p* < 0.001)
1098	υ_s_(PO_2_^–^)	DNA	NS	Reduced intensity (*p* < 0.001)	Increased intensity (*p* < 0.01)
1226	υ(C-N)	Amide III (β-sheet)	Reduced intensity (*p* < 0.001)	Increased intensity (*p* < 0.05)	NS
1301	τCH_2_	Fatty acids	Reduced intensity (*p* < 0.01)	Reduced intensity (*p* < 0.001)	NS
1437	αCH_3_,αCH_2_	Fatty acids, Sphingomyelin	Reduced intensity (*p* < 0.01)	Reduced intensity (*p* < 0.001)	NS
1442	αCH_3_,αCH_2_	Lipids	NS	Reduced intensity (*p* < 0.001)	NS
1452	δCH_2_, δCH_3_	Proteins	Increased intensity (*p* < 0.05)	NS	NS
1488	NH_3_^+^	Collagens	Increased intensity (*p* < 0.001)	Reduced intensity (*p* < 0.001)	NS
1554	υ(C = C)	Amide II	NS	NS	NS
1578	N_3_	Purines	Increased intensity (*p* < 0.001)	Increased intensity (*p* < 0.01)	NS
1662	υ_s_(C = C)	Cholesterols, Amide I (β-sheet)	Increased intensity (*p* < 0.001)	Reduced intensity (*p* < 0.01)	Increased intensity (*p* < 0.001)
1667	υ(C = C)	Amide I (α-helix), Proteins	Increased intensity (*p* < 0.001)	Reduced intensity (*p* < 0.05)	Increased intensity (*p* < 0.001)

*NS non-significant; α- scissoring, β- bending, δ- deformation, τ- twisting, υ- stretching, υs- symetric stretching. Based on: Gelder et al., 2007; Movasaghi et al., 2007; Başar et al., 2012; Talari et al., 2014; Czamara et al., 2015; Surmacki et al., 2015; González-Solís et al., 2018; Keleştemur et al., 2018; Balan et al., 2019; Casal-Beiroa et al., 2021.*

### Uvaol Prevents IL-1β and IFN-γ Secretion Triggered by Group B *Streptococcus*

**FIGURE 3:**
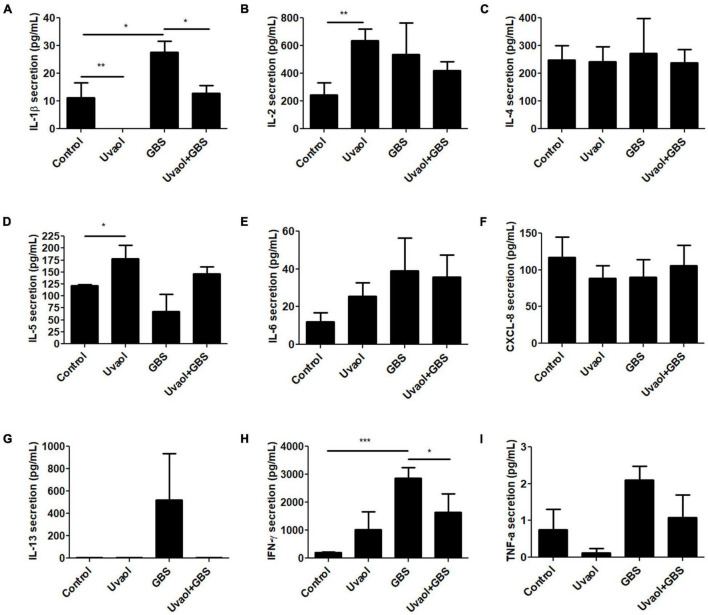
Cytokine production of trophoblast cells is altered by GBS and uvaol. HTR-8/SVneo cells were treated with 10-μM uvaol (uvaol), GBS at 1 × 10^6^ CFU, or both for 24 h, and the supernatants were analyzed for IL-1β **(A)**, IL-2 **(B)**, IL-4 **(C)**, IL-5 **(D)**, IL-6 **(E)**, CXCL-8 **(F)**, IL-13 **(G)**, IFN-γ **(H)**, and TNF-α **(I)** by flow cytometry. Bar graphs represent mean values ± S.E.M.; *n* = 6 in duplicate. *, *p* < 0.05; **; *p* < 0.01; ***, *p* < 0.001. One-way ANOVA with Tukey *post hoc* test.

Cytokine production by trophoblast cells was analyzed after 10^6^ CFU of inactive GBS or with prior uvaol treatment. IL-10 had undetectable levels in all groups. IL-1β secretion increased in the GBS group compared to Control (10.8 ± 5.3 pg/ml to 27.6 ± 4.03 pg/ml; *p* < 0.05), whereas uvaol prevented this effect (12.87 ± 2.85 pg/ml; *p* < 0.05) (Figure 3A). IL-2 secretion was unchanged by GBS, but uvaol alone increased its production compared to Control (247.2 ± 88.08 pg/ml to 636 ± 82.98 pg/ml; *p* < 0.01) (Figure 3B). IL-4 secretion was unchanged in all analyzed conditions (Figure 3C), and uvaol increased IL-5 secretion compared to Control (120.01 ± 4.56 pg/ml to 178.8 ± 27.44 pg/ml; *p* < 0.05) (Figure 3D). Regarding IL-6, CXCL-8, and IL-13, they were not affected by any treatment (Figures 3E–G). Regarding IFN-γ secretion, it was remarkably increased by GBS exposure (206.2 ± 15.72 pg/ml to 2,870 ± 371.7 pg/ml; *p* < 0.001), while, in the Uvaol + GBS group, IFN-γ secretion was partially halted in relation to the GBS group (1,640.1 ± 654.5 pg/ml; *p* < 0.05) (Figure 3H). Lastly, TNF-α levels were unchanged in all groups (Figure 3I). Therefore, GBS increased IL-1β and IFN-γ in trophoblast cells, indicating a characteristic inflammatory state in our trophoblast cell culture that was mostly prevented by the addition of uvaol.

### Uvaol Reduces Trophoblast Cell Phagocytosis and Oxidative Stress

**FIGURE 4:**
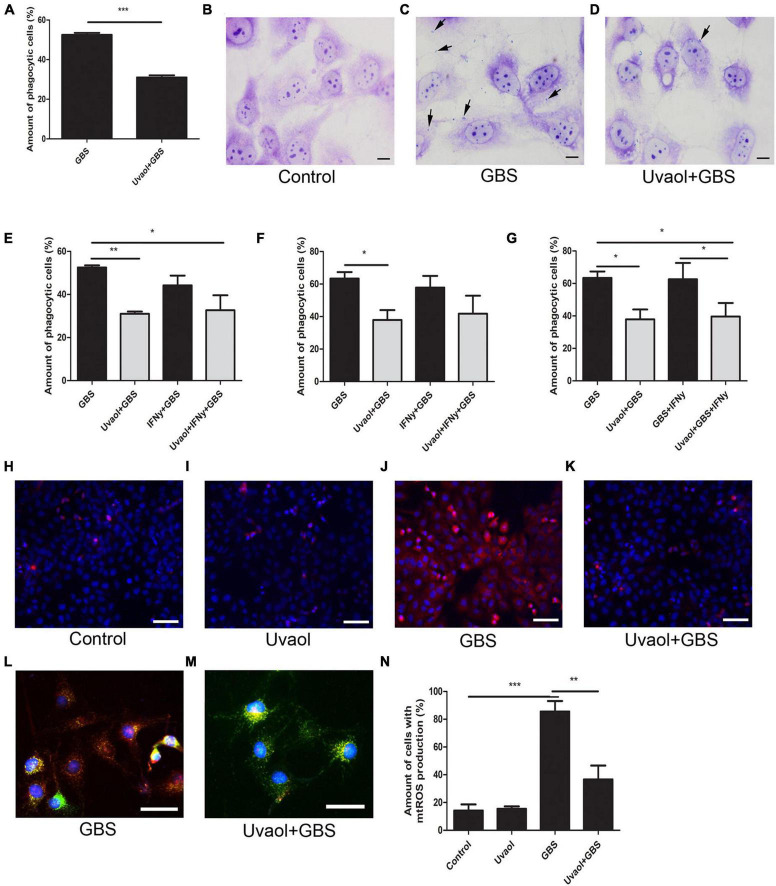
Uvaol reduces trophoblast cells phagocytosis and mtROS production. **(A)** Phagocytosis quantification after 2 h of GBS inoculation. HTR8SV/neo cells were treated with 10-μM uvaol for 1 h and incubated with GBS at 10^6^ CFU for further 2 h. A bar graph represents mean values ± S.E.M.; *n* = 4 in triplicate. ***; *p* = 0.004. Paired *t-test*. **(B–D)** Cells were stained by Giemsa staining for phagocytosis assessment, where: **(B)** control, **(C)** GBS, and **(D)** uvaol + GBS group. Black arrows show bacteria in trophoblast cells. 1,000 × magnification. Scale bars represent 20 μm. **(E)** Phagocytosis quantification after 2 h of GBS inoculation. HTR8SV/neo cells were treated for 24 h with 100 UI/ml IFN-γ and/or 1 h with 10-μM uvaol before GBS at 10^6^ CFU incubation for further 2 h. **(F)** Phagocytosis quantification after 24 h of GBS inoculation. HTR8SV/neo cells were treated for 24 h with 100-UI/ml IFN-γ and/or 1 h with 10-μM uvaol before GBS at 10^6^ CFU incubation for further 2 h. **(G)** Phagocytosis quantification after 24 h of GBS inoculation. HTR8SV/neo cells were treated for 1 h with 10-μM uvaol before GBS at 10^6^ CFU for 2 h, and 100 UI/ml IFN-γ for further 22 h. A bar graph represents mean values ± S.E.M.; *n* = 3 in triplicate. *; *p* < 0.05, **; *p* < 0.01. One-way ANOVA with Tukey *post hoc* test. **(H–K)** Cells were stained with MitoSOX red and nuclei with DAP-I, where: **(H)** control, **(I)** uvaol, **(J)** GBS, and **(K)** uvaol + GBS group. 200× magnification. Scale bars represent 200 μm. **(L,M)** Cells were stained with MitoTRACKER green, MitoSOX red, and nuclei with DAP-I were **(L)** GBS and **(M)** uvaol + GBS group. 1,000× magnification. Scale bars represent 100 μm. **(N)** mtROS quantification. A bar graph represents mean values ± S.E.M.; *n* = 3 in triplicate. **; *p* < 0.01, ***; *p* < 0.001. One-way ANOVA with Tukey *post hoc* test.

Trophoblast cell phagocytosis of GBS and oxidative stress generation was also analyzed. As a result, trophoblast cells, indeed, phagocytized the inactive GBS (Figures 4A–C). With the addition of uvaol, the number of cells that had phagocyted inactivated GBS after 2 h was greatly reduced from 52.50 ± 1.04 to 31 ± 1.08% (*p* = 0.0004) (Figures 4A–D). With the addition of IFN-γ before GBS 2-h incubation, no changes were found (Figure 4E). Analyzing the 24-h incubation of GBS, uvaol could still reduce the phagocytosis amount (63.37 ± 4% to 37.92 ± 6.06%; *p* < 0.05) (Figure 4F), and IFN-γ addition prior or after GBS incubation could not increase phagocytosis (Figure 4G). Moreover, using live GBS, trophoblast cells phagocyted much less GBS (20.55 ± 0.27%), although uvaol had a similar effect on further reduction (9.29 ± 0.57%; *p* = 0.0037) (Supplementary Figure 1). Regarding mitochondrial-derived reactive oxygen species (mtROS), it was scarcely detected in the control and uvaol groups (Figures 4H,I,N). Group B *Streptococcus* incubation dramatically increased mtROS compared to control (14.24 ± 4.41% to 85.55 ± 7.56%; *p* < 0.001), whereas uvaol was able to, at least, partially prevent mtROS production (36.62 ± 10.02%; *p* < 0.01) (Figures 4H–N).

### Uvaol Prevents Reduced Trophoblast Cell Motility Induced by Group B *Streptococcus*

**FIGURE 5:**
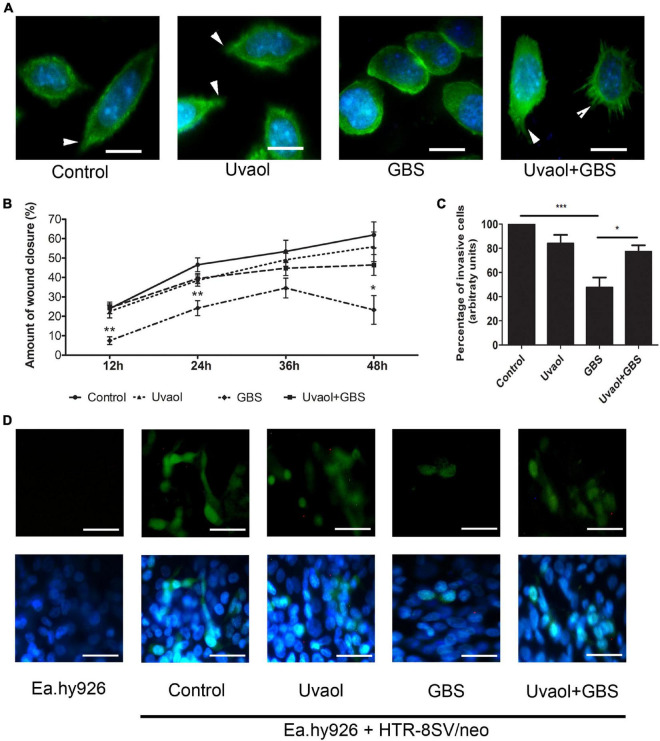
GBS reduces trophoblast cell motility, and uvaol prevents reduction. **(A)** Phalloidin (green) and nuclei staining (DAP-I) of trophoblast cells from control, 10-μM uvaol, GBS at 1 × 10^6^ CFU, or both (uvaol + GBS). Filopodia are identified by white arrowheads. 1,000× magnification. Scale bars represent 50 μm. **(B)** Percentage of *in vitro* wound closure per time point analyzed (12, 24, 36, and 48 h). A bar graph represents mean values ± S.E.M.; *, *p* < 0.05; **; *p* < 0.01. Two-way ANOVA with Bonferroni *post hoc* test. **(C)** Percentage of invasive trophoblast cells through 3D vascular invasion assay. A bar graph represents mean values ± S.E.M.; *, *p* < 0.05; ***; *p* < 0.001. One-way ANOVA with Tukey *post hoc* test. **(D)** Representative images from the 3D coculture vascular invasion assay with CFSE-stained trophoblast cells and nuclei stained with DAP-I, where: (first column) Ea.hy926 endothelial cells, (second column) control (endothelial + trophoblast cells), (third column) with uvaol, (fourth column) with GBS, (fifth column) with uvaol and GBS. 400× magnification. Scale bars represent 200 μm. All experiments were *n* = 3 in triplicate.

Phalloidin staining was performed to evaluate GBS effects on trophoblast cells. Although Control and uvaol groups were characterized by a majority of fusiform cells with filopodia and F-actin stress fibers, cells in the GBS group cells were roundish and with fewer filopodia, while uvaol treatment partially prevented these changes (Figure 5A). Trophoblast cells also had their migration evaluated with *in vitro* scratch assay for 48 h. The scratch closure rates of the Control group were 23.92% ± 2.47% after 12 h, 46.54% ± 3.52% after 24 h, 53.4% ± 5.75% after 36 h, and 61.89% ± 6.64% after 48 h (Figure 5B). Uvaol addition has remained unchanged the wound closure rate. Nevertheless, the GBS group had a significant reduction in the scratch closure at 12 h, 24 h, and 48 h compared to Control (respectively, *p* < 0.01, *p* < 0.01, and *p* < 0.05), since GBS closure rates were 7.44% ± 1.97% after 12 h, 24.2% ± 3.84% after 24 h, 34.55% ± 5.07% after 36 h, and 23.29% ± 7.38% after 48 h (Figure 5B). Uvaol addition before GBS incubation prevented the migratory reduction induced by GBS: 24.37% ± 2.99% after 12 h (*p* < 0.01), 39.44% ± 2.6% after 24 h (*p* < 0.05), 44.73% ± 3.77% after 36 h, and 46.41% ± 5.37% after 48 h (*p* < 0.05) compared to the GBS group (Figure 5B).

### Uvaol Prevents Group B *Streptococcus*-Induced Reduction of Trophoblast Invasion in a Three-Dimensional Model of Coculture With Endothelial Cells

Since trophoblast cells must invade and remodel uterine spiral arteries during pregnancy, the interface of the trophoblast-endothelial cells was analyzed with a 3D coculture vascular invasion assay. As such, control invasion was set to 100%. Uvaol unchanged invasion rates. Nevertheless, the GBS group reduced by half the number of invading cells, where only 47.54% ± 8.28% cells successfully invaded through the endothelial cells and Matrigel mixture (*p* < 0.001) (Figures 5C,D). When uvaol was added before GBS, the group had 77.22% ± 5.28% of invaded cells (*p* < 0.05), representing important prevention of GBS-induced changes (Figures 5C,D).

### Invading Trophoblast Cells Change Endothelial Cell Production of Vasoactive Molecules

**FIGURE 6:**
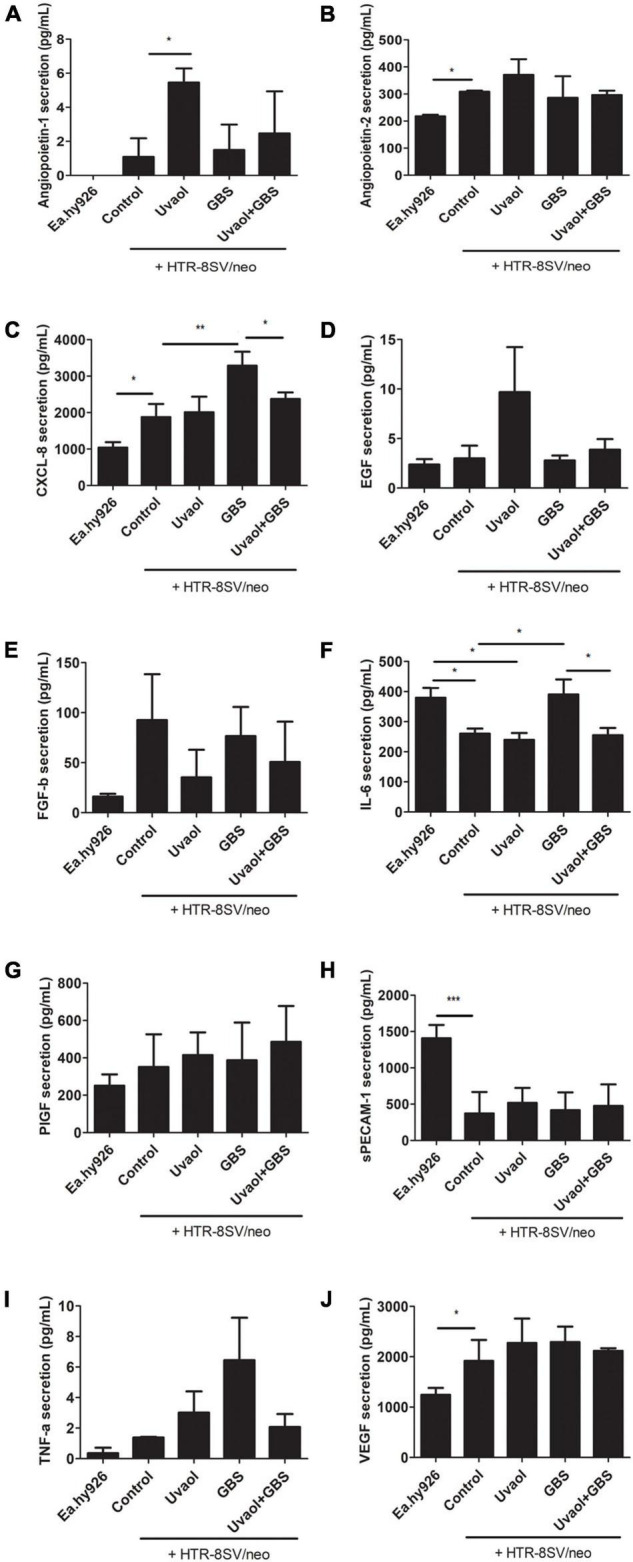
Vasoactive factors secretion from the 3D coculture vascular invasion assay. The bottom supernatants from the 3D coculture vascular invasion assay were collected and analyzed for angiopoietin-1 **(A)**, angiopoietin-2 **(B)**, CXCL-8 **(C)**, EGF **(D)**, FGF-b **(E)**, IL-6 **(F)**, PlGF **(G)**, sPECAM-1 **(H)**, TNF-α **(I)**, and VEGF **(J)** through flow cytometry. The analyzed groups were composed by Ea.hy926 endothelial cells alone, with HTR8SV/neo trophoblast cells (control), added with 10-μM uvaol or GBS at 1 × 10^6^ CFU, or both (uvaol + GBS). A bar graphs represent mean values ± S.E.M.; *n* = 3 in triplicate. *, *p* < 0.05; **, *p* < 0.01; ***, *p* < 0.001.

Endothelial cells produced almost all vasoactive factors measured here, except Angiopoietin-1 (Figure 6A). Angiopoietin-2 secretion was 217.7 ± 5.62 pg/ml, CXCL-8 secretion was 1,038 ± 149.6 pg/ml, EGF secretion was 2.37 ± 0.55 pg/ml, FGF-β secretion was 16.09 ± 2.78 pg/ml, IL-6 secretion was 379.6 ± 32.92 pg/ml, PlGF secretion was 251.1 ± 60.25 pg/ml, sPECAM-1 secretion was 1,408 ± 182.7 pg/ml, TNF-α secretion was.36 ± 0.36 pg/ml, and VEGF secretion was 1,244 ± 137.4 pg/ml (Figures 6B–J). Cocultured of trophoblast cells with endothelial cells (Control group) changed their basal production. As such, Angiopoietin-2 secretion was increased to 308.5 ± 4.33 pg/ml (*p* < 0.05), CXCL-8 was increased to 1,874 ± 360.7 pg/ml (*p* < 0.05), and VEGF was also increased to 1,915 ± 417.6 pg/ml (*p* < 0.05) (Figures 6B,C,J). Inversely, trophoblast cells addition reduced IL-6 and sPECAM-1 secretion by endothelial cells. IL-6 secretion was reduced to 260.3 ± 16.38 pg/ml (*p* < 0.05), and sPECAM-1 was remarkably reduced to 373.3 ± 293.9 pg/ml (*p* < 0.001) (Figures 6F,H). Trophoblast cells cultured under the same conditions but, without endothelial cells, did not produce detectable levels of Angiopoietin-1, while no molecules were solely changed by uvaol, and GBS incubation increased IL-6 production from 465.1 ± 40.76 to 846.3 ± 32.74 pg/ml (*p* < 0.05) (Supplementary Figure 2).

### Uvaol Prevented the Group B *Streptococcus*-Induced Increase of CXCL-8 and IL-6 in the Three-Dimensional Vascular Invasion Coculture Model

Group B *Streptococcus* (GBS) at 10^6^ CFU induced the production of inflammatory cytokines that also have vasoactive functions, such as CXCL-8 and IL-6, whereas uvaol addition completely prevented their increase. In fact, CXCL-8 secretion dramatically increased to 3,285 ± 383.5 pg/ml (*p* < 0.05, compared to Control), whereas the uvaol + GBS group had 2,372 ± 179.8 pg/ml (*p* < 0.05, compared to GBS) (Figure 6C). The same pattern occurred to IL-6, which was upregulated to 390.2 ± 49.85 pg/ml in the GBS group (*p* < 0.05, compared to Control), and reduced to 254.7 ± 24.12 pg/ml in the uvaol + GBS group (*p* < 0.05, compared to GBS) (Figure 6F). It is worth noting that trophoblast cells 2, dimensionally cultured alone with GBS, had no increase in CXCL-8 and IL-6 levels (Figures 4E,F), whereas the results with three-dimensional culture had an increase in IL-6 (Supplementary Figure 2). Although we cannot exactly affirm from which cell CXCL-8 and IL-6 increased production was derived, the 3D co-culture model was a powerful method that increased the analysis complexity and provided CXCL-8 and IL-6 as key molecules in the interaction of trophoblast cells, endothelial cells, and GBS.

### Uvaol Prevented the Group B *Streptococcus*-Induced Increase of CXCL-8 and IL-6 in Placental Explants Exposed to Group B *Streptococcus*

**FIGURE 7:**
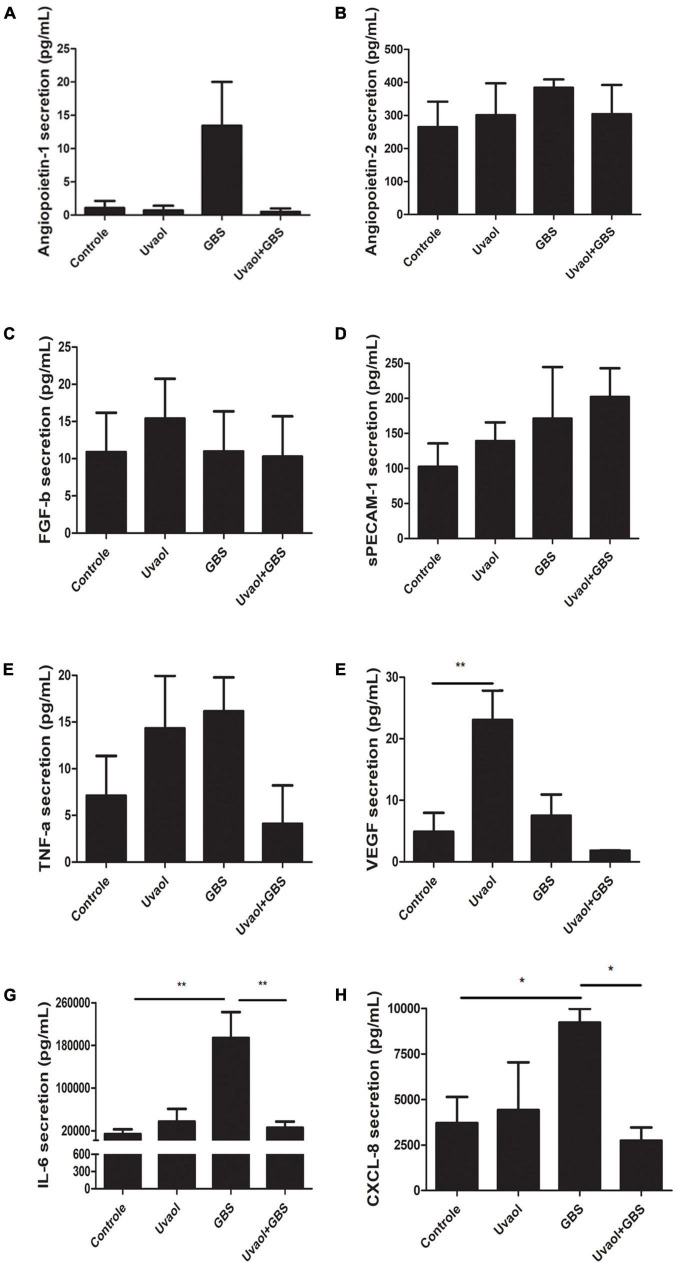
Vasoactive factors secretion by term chorionic villi explants. The supernatants from the term chorionic villi explants were collected and analyzed for angiopoietin-1 **(A)**, angiopoietin-2 **(B)**, FGF-b **(C)**, sPECAM-1 **(D)**, TNF-α **(E)**, VEGF **(F)**, IL-6 **(G)**, and CXCL-8 **(H)** through flow cytometry. The analyzed groups were composed by control, added with 50-μM uvaol or GBS at 1 × 10^6^ CFU, or both (uvaol + GBS). A bar graphs represent mean values ± S.E.M.; *n* = 5 in duplicate. *, *p* < 0.05; **, *p* < 0.01.

Placental chorionic villi explants were also analyzed for their production of vasoactive factors since their production can affect maternal-fetal blood vessels. As a result, EGF was not found in any analyzed group. Angiopoietin-1 tended to increase its production in the GBS group (Figure 7A), whereas Angiopoietin-2, FGF-b, sPECAM-1, and TNF-α had unchanged levels despite of the addition of GBS and/or uvaol (Figures 7B–E). Uvaol alone increased VEGF production compared to control (4.989 ± 3.06 pg/ml to 23.03 ± 4.8 pg/ml, *p* < 0.01), although, when with the GBS, the effect disappeared (Figure 7F). Interestingly, as the same patterns observed in our 3D vascular invasion coculture model, GBS greatly increased IL-6 production compared to control (13,800 ± 9,039 pg/ml to 194,000 ± 48,490 pg/ml, *p* < 0.01) (Figure 7G), the same occurring to CXCL-8 (3,706 ± 1,428 pg/ml to 9,220 ± 754.8 pg/ml, *p* < 0.05) (Figure 7H). As such, uvaol addition prevented the GBS-induced increase in IL-6 and CXCL-8, as IL-6 levels reduced to 25,580 ± 11,680 pg/ml (*p* < 0.01) (Figure 7G), and CXCL-8 levels reduced to 2,740 ± 719.8 pg/ml (*p* < 0.05) (Figure 7H).

## Discussion

Herein, we showed that incubation of inactivated GBS at 106 CFU on HTR-8/SVneo trophoblast cells and term chorionic villi explants have not induced changes in their viability; therefore, it was used in our *in vitro* experiments. Nevertheless, GBS incubation, either live or inactivated, is known to induce cell death in trophoblast cells (Kaplan et al., 2008; Botelho et al., 2019). Since live GBS can induce maternal death, *in vitro* studies have certain limitations of reproducibility, such as the maternal health situation, trimester of GBS infection, presence of chorioamnionitis, and its degree. As such, live GBS can lead to wide variance of clinical outcomes that are difficult to reproduce *in vitro*. To overcome such issues, we employed inactivated GBS in this study, where we could analyze the specific effects caused only by the physical contact of the bacteria to trophoblast cells, isolating infection variables. Corroborating our rationale, previous studies already showed similar results when live and inactivated GBS were tested in animal models, where placental, fetal, and offspring outcomes (chorioamnionitis, FIRS, pathological, and behavioral effects on the offspring) were alike (Allard et al., 2016, 2019; Giraud et al., 2020).

Our results with a non-lethal inactivated GBS concentration highlight the need for further studies on GBS effects on the placenta and its cells. Trophoblast cells had intense alterations in their biochemical signature, observed in Raman spectroscopy multivariate analysis using different models (PCA, HCA, and OPLS-DA). All the different analyses demonstrated their potential by separating cells into four distinct clusters that were equivalent to the groups being studied. The OPLS-DA model also found 86% of predictive accuracy on samples distinctively separated among the groups. It is important to highlight that this study is the first to use Raman spectroscopy to show the biochemical differences caused by GBS in trophoblast cells. In one of the few publications regarding Raman spectroscopy in the placenta, Chen et al. (2014) found some band intensities to be changed in preeclamptic placentas compared to healthy placentas. In their study, preeclamptic placentas had differences in bands related to amino acids and amide I, assigning the amide I difference to protein structural disorders. Also, they found unusual spectra for phenylalanine and tryptophan, which correlated with oxidative modifications common in preeclampsia (Chen et al., 2014). Interestingly, we found some similar modifications in our trophoblast cells incubated with GBS, which had altered levels of bands related to phenylalanine and amide I. Other bands related to lipids or protein conformations (amide II and amide III) were also deeply changed. For example, the reduction of 1,662 cm^−1^ intensity has been related to decreased proliferation and migration due to protein abnormalities (Wesełucha-Birczyńska et al., 2013). We also identified altered bands related to DNA, RNA, cholesterols, fatty acids, and membrane phospholipids, which could indicate changes in membrane assembly, stiffness, activity, and function (Gelder et al., 2007; Movasaghi et al., 2007; Czamara et al., 2015). The uvaol treatment before GBS incubation halved the number of bands altered by GBS only. These bands were mainly related to DNA, RNA, phenylalanine, glutathione, glycosaminoglycans, myristic acid, membrane phospholipids, cholesterols, and amide I, whereas uvaol did not affect other bands related to amide I, amide III, fatty acids, lipids, proteins, and purines. Further studies need to be performed to deeply investigate the molecular interactions and effects provoked by uvaol. Nonetheless, the antagonist effect observed on DNA and RNA-related bands was sound, protecting cells from small alterations that could lead to damage/death soon. This protection might be connected with a possible protective effect on the cytoplasmic membrane, observed by membrane phospholipids, cholesterols, glycosaminoglycans, and myristic acid-related bands. Interestingly, our previous study showed that GBS at 10^8^ CFU disrupted the cytoplasmic membrane, decreased its stiffness, and induced trophoblast cell death (Botelho et al., 2019). As such, it seems that the GBS-inducing alterations are still present here, but not in intensity to induce cell death at the time point we analyzed.

Regarding cytokines production, IL-1β and IFN-γ were increased by GBS at 10^6^ CFU, similarly to the published alterations provoked by 10^8^ CFU (Botelho et al., 2019), although IL-2 and IL-4 were not changed herein. IL-1β is a central cytokine produced in GBS response (Bergeron et al., 2013; Gupta et al., 2014), and IFN-γ is also secreted by activated CD4 + T cells in mice models of GBS infection (Chen et al., 2015). Both cytokines are also closely related to vascular dysfunctions, considered predictive and prognostic markers of vascular diseases (LaMarca et al., 2011; Raghupathy, 2013). These cytokines could be produced by indirect binding of inactive GBS to trophoblast cell membranes and by phagocytosis. The phagocytosis was accessed for live and inactive GBS, and live GBS was less phagocyted than inactive GBS, which could be possible due to evasion mechanisms of live GBS (Patras and Nizet, 2018). The GBS phagocytosis mechanisms vary widely due to 10 known capsular serotypes, and, to our knowledge, no specific mechanisms were reported to each one of these serotypes, as phagocytosis in macrophages strongly differed across clinical GBS isolates, but no correlation was found to capsular serotypes, genetic sequence types, pilus types or clinical sources (Rogers et al., 2018). Surprisingly, recombinant IFN-γ was not able to increase or restore phagocytosis in our model, as previously reported by others (Albieri et al., 2005; Tachibana et al., 2015). Regarding uvaol, it is not described in the literature to reduce phagocytosis, although a possible explanation would be the effects visualized in Raman spectroscopy related to the cell membrane and/or by membrane stiffness increase induced by this triterpene (Botelho et al., 2019).

Moreover, cells increased the production of mtROS. In several biological systems, mtROS are thought to play key roles in the adaptation to different stimuli, including hypoxia, cytokine stimulation, and calcium influx (Sena and Chandel, 2012). Although the redox biology of pregnancy and associated complications remain largely unexplored, evidence suggests that dysregulation of mtROS homeostasis causes mitochondrial dysfunction and oxidative stress, and these events are associated with the onset of adverse gynecological outcomes, mostly linked to vascular diseases (Sánchez-Aranguren et al., 2014; Karaa et al., 2019). As such, the one proposed mechanism of endothelial dysfunction is that poor trophoblast invasion is caused by increased ischemia/reperfusion and inflammation, which trigger oxidative stress from unbalanced ROS formation, generating superoxide and peroxynitrite. Both are thought to induce lipid peroxidation, tyrosine nitration, protein modifications, and DNA damage, leading to endothelial dysfunction (Sánchez-Aranguren et al., 2014). Interestingly as well, these modifications caused by superoxide production correlate with several altered bands in Raman spectroscopy results, and further studies need to be performed to better understand these correlations. An important finding was the protective effect of uvaol on preventing mtROS formation in our model. Corroborating our results, other studies also showed antioxidant effects of uvaol treatment (Allouche et al., 2010; Bonel-Pérez et al., 2020).

Additionally, we showed that trophoblast cells incubated with GBS had a reduction of filopodia and that the F-actin cytoskeleton was slightly disorganized. Cell motility was impaired as well, as, in the GBS group, cells closed the gap from the *in vitro* wound-healing assay three times slower than in control. Interestingly, trophoblast-reduced migration is one of the key events in vascular dysfunction and early-onset preeclampsia establishment (Fisher, 2015; Barrientos et al., 2017). Under physiological conditions, extravillous cytotrophoblast cells invade and remodel uterine spiral arteries, replacing endothelial cells and muscular linings of the uterine spiral arterioles and arteries. As a result, the spiral arteries diameter increases, allowing enhanced perfusion and increased metabolic uptake by the maternal-fetal interface. However, when the invasion is shallow, the mean arterial diameter stays approximately half of that of remodeled arteries from normal placentas (Fisher, 2015; Barrientos et al., 2017). Therefore, spiral arteries transform into high resistance vessels with endothelial cells not being entirely replaced by trophoblast cells, which activates immune cells and leads to vascular dysfunction (Kell and Kenny, 2016). Additionally, we used a 3D vascular invasion co-culture model where trophoblast cells could invade through endothelial cells to confirm and expand the motility impairment. The results depicted that the trophoblast invasion was remarkably affected by GBS. Other studies have also described the possibility of subclinical placental infections, increasing inflammation and ROS production, and decreasing trophoblast migration and endothelial invasion (López-Jaramillo et al., 2008; Kell and Kenny, 2016). Hence, uvaol treatment prevented migration and invasion impairment induced by GBS incubation. Recently, a study has corroborated our results, with uvaol presenting *in vivo* increase in wound healing and *in vitro*, endothelial cells and fibroblasts had their migration upregulated by uvaol treatment, which described to depend on PKA and p38/MAPK-signaling pathways in endothelial cells (Carmo et al., 2020).

To analyze if GBS could, indeed, provoke endothelial dysfunction, we analyzed the supernatants from placental chorionic villi explants exposed to GBS and from the bottom supernatants from the 3D co-culture invasion assay. Both models had the same vasoactive factors being increased by GBS: IL-6 and CXCL-8. Although IL-6 regulation of trophoblast cell migration is still debated (Champion et al., 2012; Sokolov et al., 2015), its roles in vascular dysfunction are well-established. IL-6 has increased levels in preeclamptic patients (Lockwood et al., 2008) and promotes thrombogenesis, increased expression of endothelial adhesion molecules, and vascular permeability (Roldán et al., 2003), increased vascular fibrosis, immune cell recruitment, endothelial activation, and its further dysfunction (Didion, 2017). Regarding CXCL-8, it is also increased in preeclamptic patients (Valencia-Ortega et al., 2019), and this potent neutrophil chemoattractant increases vascular permeability (Petreaca et al., 2007). It can help neutrophils infiltrate uterine spiral arteries and release ROS, MMPs, and thromboxane, increasing inflammation and presenting a pivotal role in vascular dysfunction (Pinheiro et al., 2013). Moreover, Pinheiro and collaborators (Pinheiro et al., 2013) found that increased CXCL-8 positively correlated with increased IFN-γ in preeclampsia. In this context, IFN-γ decreases trophoblast migration and placental villi outgrowths (Lash et al., 2006; Verma et al., 2018) and upregulates IL-6 and CXCL-8 (Wissen et al., 2002), involved in vascular dysfunction (LaMarca et al., 2011; Pinheiro et al., 2013; Raghupathy, 2013).

Due to the severity of GBS infection, Europe and United States recommend intrapartum antimicrobial prophylaxis based on a universal intrapartum GBS screening strategy (di Renzo et al., 2015). Although prophylaxis with antibiotics is proven effective (Vornhagen et al., 2017), their use has been associated with an increased risk of neonatal death, with a risk ratio of 1.57 (Flenady et al., 2013). Also, ampicillin treatment of GBS infection in a rat model of chorioamnionitis increased IL-1β and polymorphonuclear infiltration, which are thought to affect fetal neurodevelopment (Giraud et al., 2020). Furthermore, GBS has also proved to become resistant to several antibiotics, such as tetracycline, erythromycin, and clindamycin (Nascimento et al., 2019), which add to the need for preventive strategies. One possibility is the use of nutraceuticals, such as vitamin D, folic acid, and resveratrol, which are thought to prevent the development of pregnancy-related hypertensive disorders (Schütz et al., 2017; Fogacci et al., 2020). As such, we believe that uvaol could be a potential candidate, and further studies need to be conducted to deepen our knowledge of uvaol signaling mechanisms and *in vivo* studies as well since we understand the limitations of our study and the need to be cautious about the translation of *in vitro* to *in vivo* or clinical studies.

Furthermore, our study enhances the need for GBS tracking during pregnancy, suggesting even earlier tracking than the current recommendations, since lower concentrations of GBS that could infect women subclinically might already cause trophoblast impairment, inflammation, and vascular dysfunction. Additionally, the possibility to improve maternal and neonatal pregnancy outcomes with olive oil-derived dietary supplementation deserves attention, as diets enriched by olive oil consumption are already linked to fewer cases of preeclampsia and prematurity (Laws et al., 2009; Shen et al., 2015; Irianti et al., 2017). Therefore, it is important to highlight the salutary effects of uvaol treatment to trophoblast cells, as it seems to be a potent nutraceutical capable of avoiding deleterious effects linked to GBS exposure, presenting important anti-inflammatory, antioxidant, and pro-motility effects.

## Data Availability Statement

The raw data supporting the conclusions of this article will be made available by the authors, without undue reservation.

## Ethics Statement

The study was approved at the Federal University of Alagoas Ethics Committee, also approved by Brazilian National Ethical Committee System – Plataforma Brasil (52237915.5.0000.5013). The patients/participants provided their written informed consent to participate in this study.

## Author Contributions

ALMS was responsible for maintaining the culture, uvaol, and GBS treatments, the *in vitro* wound-healing assay, and the 3D co-culture invasion assay. ECOS was responsible for Raman spectroscopy, spectrum analysis, and PCA and HCA analysis. RMB was responsible for MTT and Annexin V/PI staining. LPGT helped with *in vitro* assays and Raman spectroscopy. ALXM helped in the individual Raman intensity peak analysis and phalloidin staining. IBACR was responsible for placental explants culture and experiments. LIMA was responsible for phagocytosis assay and helped with flow cytometry assays. AKAS were responsible for mtROS assay and the phagocytosis assay with IFN-γ. KSNP helped in cell culture, ELISA, and overall analysis. ISBT helped with live GBS culture and experiments and figures preparation. M-JA was responsible for GBS culture, titration, inactivation, and preparation to send it to Brazil. GS provided invaluable help in review and editing the manuscript. STS was responsible for Raman spectral data supervision, validation, and statistical analysis. EJSF helped in reviewing and editing this manuscript, as well as supervision of Raman spectroscopy data. KSCB was responsible for all LegendPLEX assays, including analysis and discussion in the manuscript. AUB was responsible for organizing results and data analysis, writing the original draft, and general supervision. All authors contributed to the article and approved the submitted version.

## Conflict of Interest

The authors declare that the research was conducted in the absence of any commercial or financial relationships that could be construed as a potential conflict of interest.

## Publisher’s Note

All claims expressed in this article are solely those of the authors and do not necessarily represent those of their affiliated organizations, or those of the publisher, the editors and the reviewers. Any product that may be evaluated in this article, or claim that may be made by its manufacturer, is not guaranteed or endorsed by the publisher.
