# Supplementary material for: Uvaol Prevents Group B Streptococcus-Induced Trophoblast Cells Inflammation and Possible Endothelial Dysfunction
Source: Front Physiol. 2021 Dec 3;12:766382. doi: 10.3389/fphys.2021.766382 (PMC8678414; doi:10.3389/fphys.2021.766382)
Supplement: Supplementary file 1 [file Image_1.pdf]

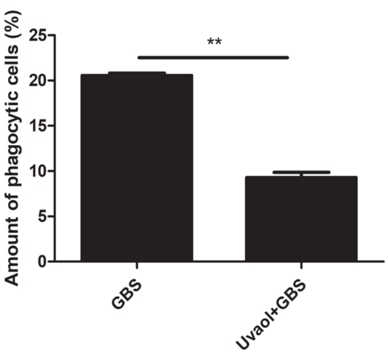

Supplementary figure 1. Uvaol reduces trophoblast cells phagocytosis of live GBS. (A) Phagocytosis quantification after 2 h of live GBS inoculation. HTR8SV/neo cells were treated with 10 μM uvaol for 1 h and incubated with GBS at 10^6 CFU for a further 2 h. Bar graph represents mean values ± S.E.M.; n = 3 in triplicates. **; p = 0.0037. Paired *t-test*.
