# Supplementary material for: Uvaol Prevents Group B Streptococcus-Induced Trophoblast Cells Inflammation and Possible Endothelial Dysfunction
Source: Front Physiol. 2021 Dec 3;12:766382. doi: 10.3389/fphys.2021.766382 (PMC8678414; doi:10.3389/fphys.2021.766382)
Supplement: Supplementary file 2 [file Image_2.pdf]

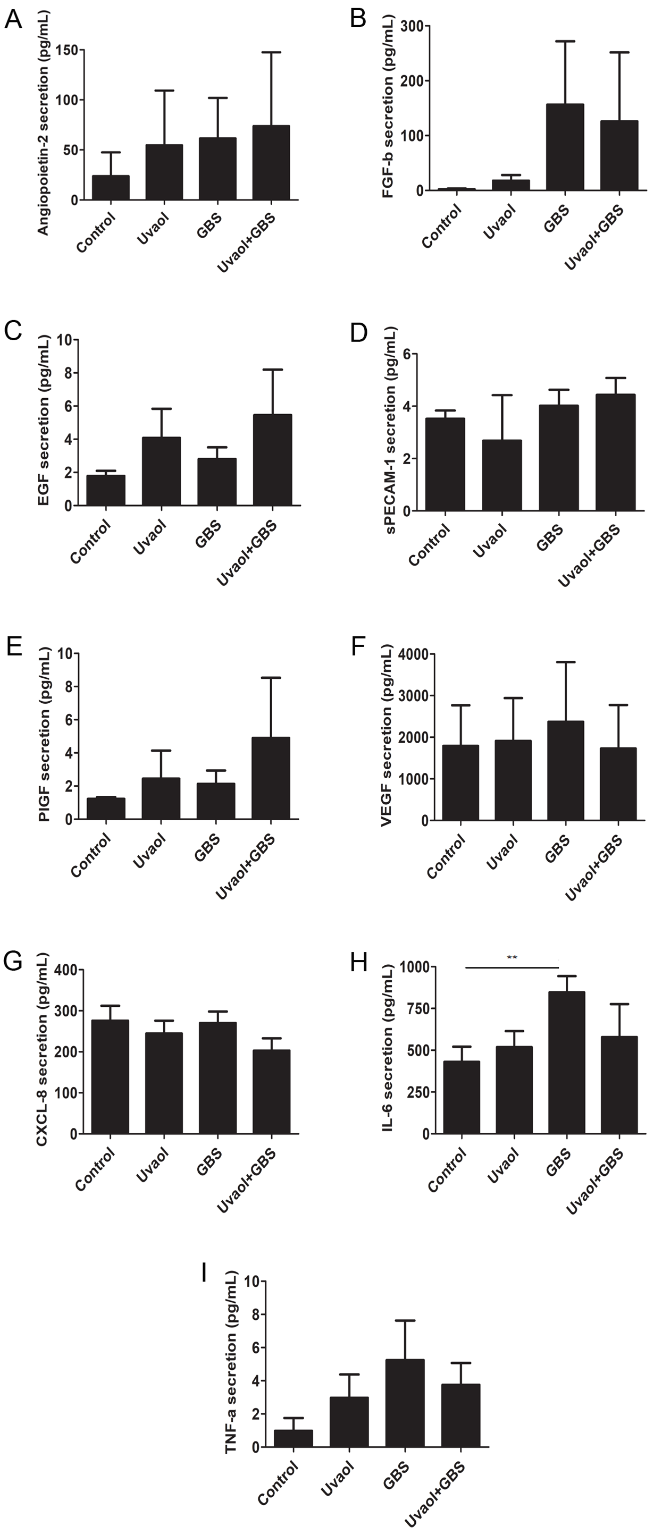

Supplementary figure 2. Vasoactive factors secretion from the 3D coculture vascular invasion assay. The bottom supernatants from the 3D coculture vascular invasion assay were collected and analyzed for angiopoietin-2 (A), FGF-b (B), EGF (C), sPECAM-1 (D), PlGF (E), VEGF (F), CXCL-8 (G), IL-6 (H), and, TNF-α (I) through flow cytometry. Angiotensin-1 was not found in the samples. The analyzed groups were composed only by HTR8SV/neo trophoblast cells alone, added with 10 µM uvaol or GBS at 10^6 CFU, or both (uvaol + GBS). Bar graphs represent mean values ± S.E.M.; n = 6 in triplicate. **, $p < 0.01$.
